# Loss-To-Follow-Up on Multidrug Resistant Tuberculosis Treatment in Gujarat, India: The WHEN and WHO of It

**DOI:** 10.1371/journal.pone.0132543

**Published:** 2015-07-13

**Authors:** Kalpita S. Shringarpure, Petros Isaakidis, Karuna D. Sagili, R. K. Baxi

**Affiliations:** 1 Department of Preventive Social Medicine, Government Medical College, Baroda, Gujarat, India; 2 Operational Research Unit, Médecins Sans Frontières, Mumbai, India; 3 The Union, South East Asia Office, New Delhi, India; The Foundation for Medical Research, INDIA

## Abstract

**Background:**

Multidrug-resistant Tuberculosis (MDR-TB) is a rising global threat to public health and concerted efforts for its treatment are diluted if the outcomes are not successful, loss to follow up (LFU) being one of them. It is therefore necessary to know the proportion and the associated reasons for LFU and devise effective patient-centered strategies to improve retention in care.

**Methods:**

A retrospective cohort study was conducted at the MDR-TB treatment site (DR-TB Site)in Central Gujarat among all patients registered from February 2010 to June 2013.LFU patients were defined as those whose treatment was interrupted for two or more consecutive months for any reason. Descriptive statistics, survival analysis and multivariate modeling were used to determine the proportion of patients LFU and to assess associations between LFU and selected demographic and clinical factors.

**Results:**

A total of 796 patients were enrolled during the study period; 71.9% were male and the median age was 35 years [Interquartile range (IQR) 27-45].The overall proportion of LFU patients was 153/796 (19.2%).The majority of LFU patients (133/153 i.e.87%) were lost within the first 6 months of treatment. Ambulatory treatment initiation (adjusted Hazards ratio aHR=2.63, CI:1.01-6.86), different providers in IP and CP ( aHR=1.27, CI:1.18-1.38)and culture conversion after more than 4 months of treatment(aHR=1.34, CI: 1.21-1.49)were found to be significantly associated with LFU in multivariate models.

**Conclusions:**

A high proportion of LFU among patients on MDR-TB treatment was found in a programmatic setting in India. Clinical but equally important programmatic factors were associated with LFU, accounting for one-fifth of all the outcomes of MDR-TB treatment. Proper training for DOT providers and aggressive counseling and health system strengthening with patient friendly follow up services may help reduce LFU.

## Introduction

Multidrug-Resistant Tuberculosis (MDR-TB) is a significant and complex public health problem in a number of countries and an obstacle to effective Tuberculosis (TB) control, given the long duration of treatment, adverse events and unsatisfactory treatment efficacy in programmatic conditions [1,2]. Almost 60% of the global MDR-TB cases are in India, China and the Russian Federation. The largest increase of MDR-TB cases between 2011 and 2012 were in India, South Africa and Ukraine [3]. India alone accounted for 64,000 MDR-TB cases out of 300,000 MDR-TB cases estimated globally to occur among the notified pulmonary TB cases annually[4]. In the State of Gujarat, 4321 out of 24619 notified TB cases were confirmed as MDR/Rifampicin Resistant TB and out of them, a total of 3344 were initiated on treatment [5].

Despite early diagnosis and treatment initiation of MDR-TB, mortality and loss to follow up (LFU)remain high in Gujarat at 20% each[4]. This proportion of LFU becomes a major challenge in the treatment of MDR-TB due to irregular treatments and increased costs. LFU patients experience increased risk of recurrence and TB-related deaths because they are more likely to remain sick and infectious, endanger their own life and continue spreading the resistant strains through airborne transmission[6].

Systematic reviews have described a plethora of characteristics associated with LFU along with other poor TB outcomes while on TB treatment, which include socio-demographic factors (age, male sex, lower socioeconomic status, low education level), clinical factors(low BMI, alcoholism, HIV-infection, drug use) and programmatic factors(treatment adverse events, prior history of anti-tuberculosis treatment, previous LFU, poor bacteriologic response and treatment duration prior to LFU)and certain other factors such as lack of social support, dissatisfaction with health care worker attitudes and lack of counseling[1,2,7,8,9]. Previous studies that examined risk factors specific to MDR-TB treatment LFU, found that it was most strongly predicted by substance abuse, dissatisfaction with healthcare worker’s attitudes, low or unstable socioeconomic status and poor response to treatment[7,10]. There is however, paucity of literature for programmatic factors associated with LFU in the global and Indian contexts. This study aimed to determine the proportion of LFU and the factors associated with LFU among patients put on MDR-TB treatment in a programmatic setting in Central Gujarat, India. A comprehensive understanding of these factors may assist to develop effective programmatic, patient-centered strategies to improve retention in care.

## Methods

### Ethics

The study was approved by the Institutional Ethics Committee for Human Research (IECHR) of Baroda Medical College (Vadodara, India) and the Ethics Advisory Group of the International Union Against Tuberculosis and Lung Disease (Paris, France). As this was a study of routinely collected monitoring data, informed consent from the patients was not obtained. The named ethics committees specifically approved the study and waived the need for consent.

### Study Design

This was a retrospective cohort study based on record review of patients enrolled on MDR-TB treatment from February 2010 to June 2013.

### Study Setting/Area

The Drug Resistant TB (DR-TB) Site in Shree Sayaji General Hospital (SSGH), Vadodara, the second DR-TB Site in the state of Gujarat, has been established in February 2010 for initiation and monitoring of patients put on MDR-TB treatment. It receives inflow of patients from Vadodara (rural, urban and tribal), Narmada, Bharuch, Panchamahal, Anand and Dahod districts. Patients diagnosed with MDR-TB are initiated on a standardized regimen of second line drugs for 24 to 27 months (first 6–9 months of Intensive Phase and 18 months of Continuation Phase)[11].

### Study Population

All patients registered at the DR-TB Site, Vadodara from February 2010 to June 2013 were included in the study.

### Data sources and data collection

Routinely collected programmatic data were taken from the DR-TB register maintained at the DR-TB Site at Vadodara. Updated information was collected from the DR-TB Treatment cards available with the DR-TB Supervisor for data triangulation. Reasons for LFU as documented in the TB Treatment cards, the DR-TB Register and the DR-TB meeting reports were analyzed. Socio-economic status (based on below poverty line cards) was assessed on the basis of data available from the Medical Records of the department. Data collection and entry was done between January–April 2014 using a structured data capture instrument.

Explanatory variables to identify the factors associated with lost to follow up among MDR-TB patients included age, sex, socioeconomic status (based on whether they had the Below Poverty Line card or not), previous history of anti-tuberculosis treatment (ATT), HIV status, phase of treatment- (Intensive or continuation), adverse drug events and culture conversion. Main o*utcome variable* was loss to follow up. LFU patients were defined as those whose treatment was interrupted for two or more consecutive months for any reason[11]. Other MDR-TB outcomes included treatment completed, cured, failure, treatment after default, transferred out—defined as per Programme Management of DR-TB (PMDT) guidelines [11].

### Data Analysis

The forms were checked for errors and corrected on the same day of data collection. Double data entry, data cleaning and validation was done in EpiData 3.1. Patient characteristics were summarized using descriptive statistics. Proportions of patients LFU on MDR-TB Treatment was calculated from the number of patients who were LFU out of the total number of patients registered in the study cohort.

Patients who were LFU from treatment were compared with those who died, were transferred out of the program, were cured, or experienced treatment failure. Student’s t-test, Chi-square or Fisher’s exact test was used to assess differences of variables between groups, as appropriate. Uni-variate and multi-variate analyses to assess risk factors associated with LFU were done. Hazard Ratios (HR) and adjusted HR with 95% Confidence Intervals were calculated using uni-variate and multi-variate Cox regression models respectively. The time-to-LFU was determined using Kaplan-Meier analysis (data were censored by 31^st^ March 2014). All variables were included in the multi-variate proportional hazard model. A p-value of less than 0.05 was considered to indicate statistical significance. EpiData software for data entry and analysis (version 3.1 for entry and version 2.2.2.182, EpiData Association, Odense, Denmark and Epi Info7.1.3.0(EpiInfo 7/CDC) for analysis were used.

## Results

### Characteristics of the study population

Almost three-fourths of the patients were male (571/794)(72%) and the median (IQR) age of the study cohort was 35 (27–45) years. The median (IQR) weight of patients was 39 (34–45) Kg. Of them 44.3% (352/794) lived in rural areas and 77.3% (614/794) were living below the poverty line (Table 1). Approximately half of the patients (45.8%, 362/794) had taken anti-tuberculosis treatment previously, with 16.4% (44/268) having taken more than 2 years of treatment. The median duration of previous anti-tuberculosis treatment taken was 17 months (IQR 3–17 months).

**Table 1:** Baseline characteristics of MDR-TB patients on treatment, LFU and non-LFU patients at Drug Resistant TB Centre in Vadodara, Gujarat, India.

Variables	Entire Cohort*	LFU*	Non-LFU*
	N(%)	N(%)	N (%)
**Age (years) Median (IQR)**	35 (27–45)	35 (28–45)	35 (27–45)
**Weight (kg) Median (IQR)**	39 (34–45)	40 (35–45)	39 (34–45)
**Male Sex**	571/794 (71.9)	114/153 (74.5)	457/641 (71.3)
**Rural residence**	352/794 (44.3)	64/153(41.8)	288/641 (44.9)
**Below poverty line**	614/794 (77.3)	120/153 (78.4)	494/641 (77.1)
**Previous TB treatment**	362/794 (45.8)	78/153 (51.3)	284/641 (44.3)
**Duration of previous treatment >2 years**	44/268 (16.4)	52/59 (88.1)	37/209 (17.7)
**Same DOT Provider in IP and CP**	408/507 (80.5)	23/153 (15.0)	385/479 (80.4)
**HIV infected**	10/793 (01.3)	01/153(0.7)	9/640 (01.4)
**Chest X-Ray Bilateral involvement**	610/716 (85.2)	114/132(86.4)	496/584 (84.9)
**Chest X-ray Cavitation**	343/716 (47.9)	58/132(43.9)	285/584 (48.8)
**Ambulatory initiation of treatment**	65/794 (08.2)	20/153(13.1)	45/641 (07.0)
**Adverse events in IP**	151/793 (19.0)	30/153(19.6)	121/640 (18.9)
**Adverse events in CP**	34/793 (04.3)	01/20(05.0)	33/640 (05.2)
**Culture conversion time(days) Median (IQR)**	105 (93–147)	153.75 (104–180.7)	105.5 (93–149.5)
**Duration of MDR treatment before outcome(days) Median (IQR)**	361 (137–739)	142 (63.5–296)	531 (229–759.75)

*Number varies according to data available from records IQR-Inter Quartile Range; DOT-Direct Observed Treatment; IP- Intensive Phase; CP-Continuation Phase.

Of the total who continued treatment from IP to CP, 80.5% (408/507) had the same DOT provider in both treatment phases (Table 1).

### Treatment outcomes

The outcomes of all the patients put on treatment from 2010–2013 as well as the cohort with final outcomes (February 2010-March 2012) are shown in Table 2. During the study period, 796 patients were put on a standardized DR-TB regimen based on their weight. As shown in Table 2, out of the total cohort (N = 796), 11.3% were cured, 21.6% of the patients died, 19.2% were LFU, 3.3% failed treatment and were switched to a regimen for extensively drug-resistant TB (XDR-TB) treatment, 3.1% each were declared treatment completed and treatment failure and 1% were transferred-out, while 37.1% of the patients were alive and on treatment at the end of the observation period. Median (IQR) time taken for culture conversion was 153.75 (105–180.70) days among the LFU group. Among LFU patients 19.6% of had adverse events (AEs) during the Intensive phase. There were no patients whose treatment was stopped due to AEs (Table 1).

**Table 2:** MDR-TB treatment outcomes of patients at at Drug Resistant TB Centre in Vadodara, Gujarat, India.

Outcome	Entire cohort	Cohort with final outcomes declared
	N (%)	N (%)
**Cured**	90 (11.3)	90 (28.0)
**Treatment completed**	25 (03.1)	25 (07.8)
**Loss to follow up**	153 (19.2)	68 (21.1)
**Death**	172 (21.6)	87 (27.0)
**Treatment failure**	25 (03.1)	24 (07.5)
**Switched to XDR-TB treatment**	26 (03.3)	12 (03.7)
**Still on treatment**	295 (37.1)	14 (04.3)
**Treatment stopped due to ADR**	00	00
**Total**	**796** *	**322#**

*Transferred out and not recorded 10

^#^Not recorded 2

Among patients with final outcomes (n = 322), success rate was 35.7% (28% cured and 7.8% were declared treatment completed.

### LFU from treatment and time-to-LFU

**Fig 1:**
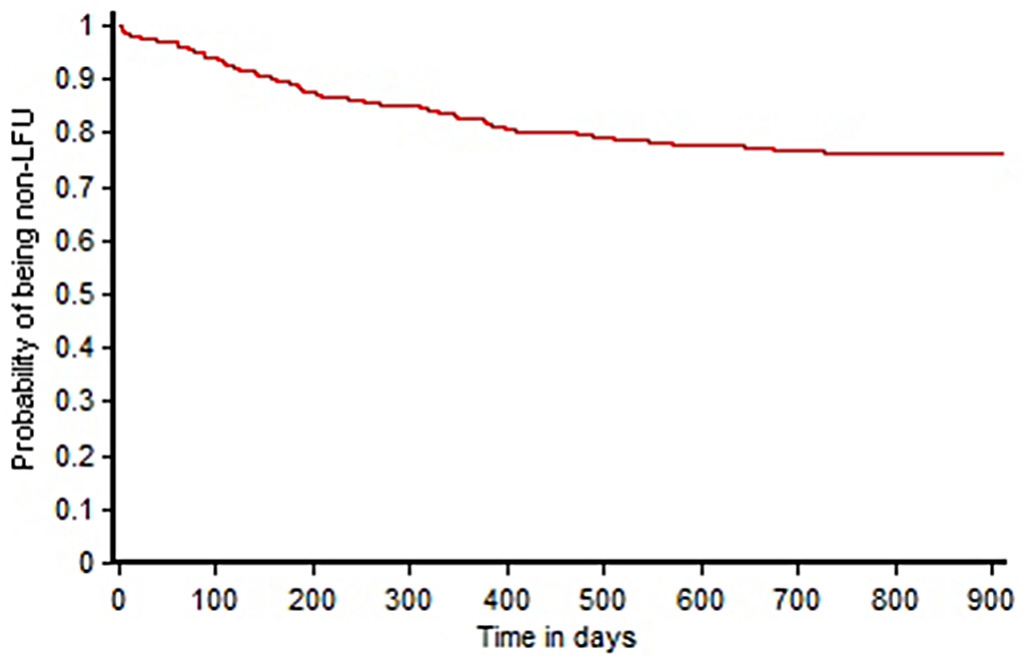
Time to LFU following initiation of MDR-TB treatment at the Drug Resistant TB Site at Vadodara, Gujarat, India using Kaplan-Meier’s analysis.

One hundred and fifty three of the 796 patients (19.2%) were LFU during the study period. The majority of the patients (86.9%, 133/153) were LFU in the first 6 months of treatment. The LFU rates were similar with each subsequent year of enrolment. The year-wise proportions of LFU patients to the year-wise cohort were 23.3%, 18.3% and 20.6% for the year 2010, 2011 and 2012 respectively. Fig 1 shows the time to LFU for patients on MDR-TB treatment using Kaplan-Meier (KM) analysis.

### Risk factors for treatment LFU

Out of 153 LFU patients, LFU was more common among males (74.5%) and patients from rural areas (41.8%). Of patients who had started ambulatory treatment at the district level (without initial hospitalization), 13.1% were LFU (Table 1).

**Fig 2:**
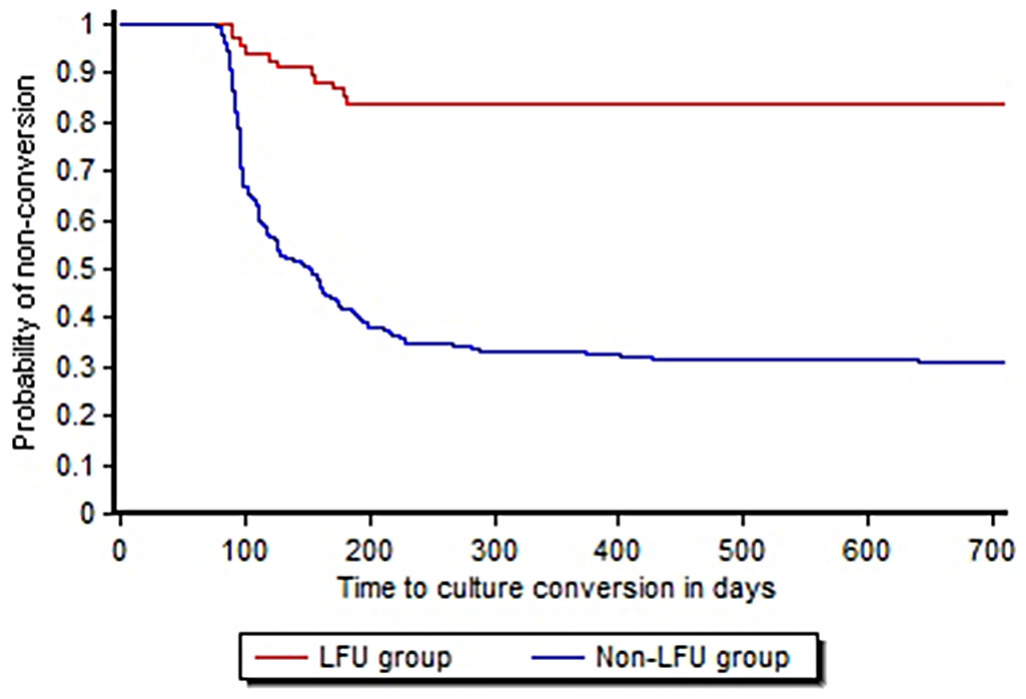
Time to culture conversion among LFU and non-LFU groups using Kaplan-Meier’s analysis.

Uni-variate analysis (Table 3)in the subset of patients from the cohort of February 2010-March 2012, whose final outcomes were declared, showed prior anti-tuberculosis treatment (p = 0.01), having different DOT provider in Intensive Phase (IP) and Continuation Phase (CP) (p<0.001) and time taken for culture conversion of more than four months (p<0.001) to be significantly associated with LFU. ‘Non-conversion’ was more common among LFU than among patients in the non-LFU group matched at similar time, as shown in the KM curve of culture conversion among the LFU group and the non-LFU group (Fig 2). In multi-variate proportional hazard models (Table 3),ambulatory treatment initiation(adjusted Hazards ratio (aHR) = 2.63, CI:1.01–6.86), different providers in IP and CP(aHR = 1.27, CI:1.18–1.38)and culture conversion after more than 4 months of treatment(aHR = 1.34, CI: 1.21–1.49)were found to be significantly associated with LFU.

**Table 3:** Factors associated with LFU among MDR-TB patients on treatment at Drug Resistant TB Centre in Vadodara, Gujarat, India.

Baseline Variables	Hazard Ratio	(95% CI)	Adjusted Hazard Ratio	(95% CI)
**Age >35 years**	0.99	0.91–1.08	0.98	0.78–1.23
**Weight>45 kgs**	0.89	0.77–1.04	1.19	0.87–1.61
**Male Sex**	1.14	0.86–1.50	1.08	0.49–1.67
**Rural residence**	0.95	0.83–1.08	1.02	0.76–1.38
**Living below poverty line**	0.97	0.74–1.27	0.85	0.49–1.47
**Previous TB treatment**	**0.99**	**0.991–0.997**	0.92	0.83–1.02
**Chest X-ray Bilateral Involvement**	1.03	0.95–1.11	0.88	0.57–1.36
**Chest X-Ray Cavitation**	0.99	0.92–1.08	1.08	0.72–1.64
**Ambulatory initiation of treatment**	1.68	0.62–4.55	**2.63**	**1.01–6.86**
**Different DOT Provider in IP and CP**	**1.15**	**1.11–1.19**	**1.27**	**1.18–1.38**
**Culture conversion time more than 4 months**	**1.10**	**1.06–1.15**	**1.34**	**1.21–1.49**
**No Adverse events in IP**	0.75	0.55–1.00	1.13	0.61–2.09
**No Adverse events in CP**	0.99	0.65–1.49	2.63	0.35–19.95

## Discussion

In this relatively large programmatic cohort of patients on MDR-TB treatment similar trends of loss-to-follow-up were seen over a four years period. There were near one-fifth of patients who were LFU. This finding is consistent with the annual LFU rates of approximately 20% LFU among patients on MDR-TB treatment in India, as per data from the Government’s Revised National TB Control Programme [4]. Systematic reviews observed a much lesser proportion of LFU i.e. 12% and 13% in countries outside Asia [1,2,12], while an individual patient data meta-analysis reported 23% LFU rates [13]. Three-fourths of the patients in this Indian cohort were males. Various other studies have shown a male predominance for the disease, accounting for almost the same proportion as in this study [8,14,15]. A study by Datta et al had reported56% of the cohort to be males [16]. The number of male patients being diagnosed and put on treatment may reflect gender discrimination or differences in health-seeking behavior in the community. Men in India are likely more widely exposed to people with infectious TB, as a consequence of their greater social interaction outside the home. Risk factors such as smoking, alcoholism and high mobility/migration are also more common among males in this context and are often associated with TB infection and active TB.

One-third of the LFU patients were between 35–44 years as previously shown [12,15]. LFU is common in this age group probably due to the responsibility of earning a daily living by doing labor work or migration for employment. This makes it more challenging to adhere to the 24–27 months long treatment [17]. There were only 6 patients below the age of 15 years, with minimum age being 10 years. Only a single patient <15 years was LFU. Very limited data exist about the prevalence of MDR-TB in the young children and adolescents, let alone the LFU in this age group. The younger children may face more challenges in being diagnosed for MDR-TB and adhering to the treatment [18]. Almost half of the LFU patients were from rural areas. Unlike prior studies, which showed that geographically isolated patients are more likely to be lost from care; we found no association between rural residence and LFU. Three fourths of the LFU patients were living below poverty line. As previously reported, socially disadvantaged groups are more likely to be lost from care than patients living in urban centers, employed and having a regular income [17,19].

HIV and MDR-TB co-infected patients were few in this cohort and only one of them being LFU prevented us from exploring the association between HIV status and LFU. However, as suggested by a study in Mumbai [20] the challenges remaining in care among co-infected patients are enormous and specific interventions are needed for this vulnerable group.

A study on a 7 year pilot experience of MDR-TB treatment in India showed that the majority of LFU patients had migrated and half of them were LFU in the first six months of treatment, which is consistent with the findings of this study [16]. We hypothesize that this is due to the fact that the early phase of treatment, which includes daily injection, is painful and accompanied by severe adverse events without providing early and significant symptom relief and clinical improvement. Qualitative studies have shown that the treatment may be experienced as “worse that the disease itself” [20]. We also believe that inadequate counseling on the duration of injectable phase and total duration, on adverse events and lack of adequate emotional and psychosocial support of patients and their families may have contributed to LFU in this cohort, especially in the early difficult phase of treatment. “Our study revealed several factors associated with LFU and we believe that the most interesting of them, from an operational point of view, were the following: (1) culture conversion after 4 months of treatment (2) ambulatory treatment initiation and (3) different DOT provider in IP and CP.

Culture conversion after 4 months, entails extending the IP (injectable phase); further increasing this difficult phase. Thus culture conversion time more than 4 months is more likely to cause LFU. It is also possible that LFU patients may be less adherent early in treatment and therefore more likely to have late culture conversion. A minor proportion had stopped treatment due to AEs, as in previous study on DR-TB experiences.[17]. However, though not statistically significant in our analysis, AEs may indirectly lead to poor treatment adherence and increase the probability of patients being LFU. A patient-centered approach may help to get a better understanding of these factors (delayed culture conversion, poor adherence and AEs being inter-related) including qualitative enquiries.

In the same context, starting ambulatory treatment without hospitalization led to LFU due to lack of one-one counseling by the DR-TB team, less knowledge imparted about the AEs which may be faced while on treatment as well as less idea of the total duration of treatment. All of the above, counseling, imparting disease knowledge and information is more effective when the patients are admitted at the DR-TB site for treatment initiation.

Our study, being part of an operational research project, focused specifically on exploring programmatic factors associated with LFU and one of the most interesting findings was that different providers in IP and CP were more likely to lead to LFU. Thus, having the same DOT provider in the IP and CP had a significant protective effect in terms of retention in care. We hypothesize that having the same provider for longer period facilitated the development of rapport and an empathetic provider-patient relationship. This may have also allowed for more personalized, tailored counseling to the patient while on treatment. Changes in DOT provider, i.e. having different DOT providers in IP and CP, more often due to the unsatisfactory attitude of health workers has been earlier associated with LFU [7]. We are currently analyzing qualitative data from in-depth interviews and group interviews collected during this study, and we believe that once completed we will be able to discuss in details such an interesting programmatic finding.

Few studies have mentioned association between alcohol abuse and LFU [2,7,8,12,19]. We were unfortunately not able to study this factor in our cohort. Gujarat being considered a “Dry” state i.e. use of alcohol is banned in the state; the variable “alcohol use” was not included in the patient files. However, we believe that there is scope for looking into all factors for LFU.

Since this was a record review based study, there were incomplete data which is one major limitation. BPL cards, which were taken as a proxy for the socio-economic status may not have given a correct picture of the same. Poverty may not have shown as a predictor of LFU in the analysis purely because a vast difference between the ‘ estimated’ and ‘identified’ poor is prevalent [21]. The number of people who are actually poor and using BPL cards is actually small. Lack of substantial numbers of children and HIV patients in the study cohort is one of the limitations of this study. Retrospective record reviews may not be the best design in studying programmatic or patient related factors for LFU-other than those mentioned on the records. However, the whole cohort was included in this study and we believe that this is a cohort representative of the region. The study followed the STROBE guidelines with robust cross-checking of the available data sources.

## Conclusion and Recommendations

LFU accounted for one-fifth of all outcomes of MDR-TB treatment in a Western Indian cohort. Ambulatory initiation of treatment, late culture conversion and not having the same DOT provider throughout the treatment were independently significantly associated with LFU. Strategies to improve provider patient relationships, training of DOT providers, aggressive counseling and evaluation of the impact of counseling may help to reduce LFU. Emphasis should be given on the need for patient-centered policies and practices.
